# Six Sigma research in healthcare is associated with global health, research and development indicators: A scientometrics study based on countries by income groups

**DOI:** 10.23938/ASSN.1124

**Published:** 2025-09-25

**Authors:** Estefany Romero-Freile, Darwin Ortiz-Cerchar, David A. Hernández-Páez, Ornella Fiorillo-Moreno, Yelson Alejandro Picón-Jaimes, Tulia Beltrán-Venegas, Johana Galván-Barrios, Ivan David Lozada-Martinez

**Affiliations:** 1 Universidad de la Costa Barranquilla Colombia; 2 Center for Meta-Research and Scientometrics in Biomedical Sciences Barranquilla Colombia; 3 Clínica Iberoamerica Barranquilla Colombia; 4 Clínica El Carmen Barranquilla Colombia; 5 Universitat Ramon Llull Facultat de Ciències de la Salut Blanquerna Barcelona Spain; 6 Universidad de la Costa Department of Health Sciences Biomedical Scientometrics and Evidence-Based Research Unit Barranquilla Colombia

**Keywords:** Total Quality Management, Quality of Health Care, Bibliometrics, Publications, Meta-Research, Gestión de la Calidad Total, Calidad de la Atención de Salud, Bibliometría, Publicaciones, Meta-Investigación

## Abstract

**Background:**

Six Sigma is widely implemented in healthcare to enhance efficiency, minimize medical errors, and improve patient safety. However, the global distribution and impact of Six Sigma research in healthcare remain underexplored. This study conducts a scientometrics analysis of Six Sigma research in healthcare, examining its association with global health, research, and development indicators across income groups.

**Methodology:**

A mixed-methods scientometrics study was employed, utilizing data from Scopus, PubMed, and other databases. Regression models and meta-analyses were applied to evaluate associations between Six Sigma research productivity and global health, research and development indicators. Publications were categorized by World Bank income groups, and bibliometric parameters such as impact were analysed.

**Results:**

A total of 804 Six Sigma-related publications in healthcare were identified, with high-income countries contributing 70.8% of the total output. The number of publications was significantly associated with adult mortality reduction in high- and upper-middle income countries (p < 0.01). Research and development expenditure showed a strong positive correlation with Six Sigma research output across all income groups. However, low-income countries exhibited minimal research activity, with no significant associations detected.

**Conclusions:**

Six Sigma research in healthcare is predominantly concentrated in high-income countries, with increasing but uneven growth in upper-middle and low-middle income countries. The limited engagement of low-income countries underscores a critical research gap.

## RESUMEN

**Fundamento:**

Six Sigma se ha implementado ampliamente en el ámbito de la salud para mejorar la eficiencia, reducir los errores médicos y fortalecer la seguridad del paciente. Sin embargo, la distribución global y el impacto de la investigación sobre Six Sigma son poco explorados. El objetivo es analizar la investigación en Six Sigma aplicada a la salud, y su relación con indicadores globales de salud, investigación y desarrollo según los niveles de ingreso.

**Métodos:**

Estudio cienciométrico de métodos mixtos, basado en datos obtenidos de Scopus, PubMed y otras bases de datos. Se aplicaron modelos de regresión y meta-análisis para examinar la relación entre la productividad en investigación sobre Six Sigma y los indicadores globales de salud, investigación y desarrollo en países de ingresos altos (PAI), ingresos medio-altos (PIMA) y bajos (PBI).

**Resultados:**

Se identificaron 804 publicaciones relacionadas con Six Sigma en el ámbito de la salud, 70,8% aportadas por PAI. El número de publicaciones se asoció con la reducción de mortalidad en adultos en PAI y PIMA (p < 0,01). El gasto en investigación y desarrollo mostró una fuerte correlación positiva con la producción científica en Six Sigma en todos los grupos de ingresos. Los PBI registraron una actividad investigativa mínima, sin detectarse asociaciones significativas.

**Conclusiones:**

La investigación sobre Six Sigma en salud se concentra predominantemente en los PAI, con un crecimiento progresivo pero desigual en los PIMA y países de bajos y medianos ingresos. La escasa participación de los PBIs evidencia una brecha crítica en la investigación.

## INTRODUCTION

Healthcare systems worldwide face persistent challenges in ensuring high-quality care, patient safety, and operational efficiency^1^. To resolve these challenges, Six Sigma -a data-driven methodology originally developed in the manufacturing sector- has emerged as a valuable approach for optimizing healthcare processes, minimizing errors, and improving patient outcomes^2^. By focusing on reducing process variability and eliminating inefficiencies, Six Sigma has been instrumental in enhancing clinical workflows, diagnostic accuracy, and healthcare delivery performance^2^. Given its widespread adoption in quality improvement initiatives, investigating the role of Six Sigma in healthcare is imperative for understanding its contribution to patient safety, cost reduction, and system-wide efficiency^3^.

The application of Six Sigma in healthcare spans various domains, including hospital management, surgical procedures, laboratory diagnostics, and medication administration^4^. Studies have demonstrated that Six Sigma methodologies can significantly decrease medical errors, streamline operational workflows, and enhance resource utilization^5^. However, despite its demonstrated benefits, research on Six Sigma’s global impact remains fragmented and often confined to localized studies. There is a need for a comprehensive evaluation of Six Sigma research trends in healthcare, particularly in the context of global health indicators and research and development investments.

Scientometrics analyses provide a powerful tool for systematically assessing the evolution, distribution, and impact of research in healthcare^6^. By leveraging bibliometric data, these analyses can uncover disparities in research productivity across countries, explore associations between scientific output and economic classifications, and highlight key trends in publication patterns^7^. Moreover, such an approach enables the identification of knowledge gaps and research opportunities, informing policymakers and healthcare stakeholders about the effectiveness of Six Sigma interventions across diverse healthcare settings^8^.

This study aims to bridge this knowledge gap by conducting a comprehensive scientometrics analysis of Six Sigma research in healthcare, stratified by World Bank income groups. The study evaluates the association between Six Sigma-related research output and key global health, research and development, and economic indicators. By adopting this approach, the findings contribute to a deeper understanding of how Six Sigma research aligns with global health priorities, offering insights into its role in shaping healthcare policies, improving healthcare quality, and advancing scientific innovation in resource-limited settings.

## METHODS

### Study design

Mixed-methods scientometrics study. The mixed-methods approach was supported by the combination of scientometrics methods (scientific publications analyzed through a bibliometric analysis) and the integration of health metrics and evaluation analysis (health econometrics)^9^^-^^12^. This study was reported following the recommendations of the BIBLIO guideline (Guideline for Reporting Bibliometric Reviews of the Biomedical Literature), which provides standards for reporting scientometrics/bibliometric studies^13^.

The study was approved by the Scientific Committee of Universidad de la Costa (Barranquilla, Colombia) (code GRA.2021-07-002-19). However, no humans, animals, or medical records were used as units of analysis.

### Data sources

An exhaustive and systematic search was conducted across various academic, including Scopus, PubMed/MEDLINE, the Web of Science Core Collection, the SciELO Citation Index, and the KCI-Korean Journal Database. These sources were selected due to their extensive global reach and substantial repository of bibliographic and citation data within the medical and health sciences. Furthermore, their stringent inclusion criteria for peer-reviewed journals ensure high-quality indexing, enhancing their credibility and reliability compared to other available sources. The justification of utilizing these databases for research of this nature has been previously validated, reinforcing their methodological rigor and reproducibility^14^^-^^18^.

### Search strategy

A structured search strategy was developed by incorporating MeSH terms and their corresponding synonyms to retrieve peer-reviewed literature that examines, explores, or synthesizes evidence related to Six Sigma in healthcare. This methodology prioritized systematically indexed publications across multiple bibliographic databases, encompassing fields such as medicine, nursing, dentistry, health professions, biochemistry, genetics, molecular biology, immunology, neuroscience, pharmacology, toxicology, and pharmaceutical sciences. During the initial phase, pilot tests were conducted by integrating diverse terms and indexing tags across various search engines and databases to refine and enhance the search strategy. The optimized version, which yielded the most precise and relevant results when applied in the Scopus database is shown in Appendix I; this strategy was adapted for use in each of the other databases or search engines.

The search was carried out on July 30, 2024, in both English and Spanish. The preliminary screening of titles and abstracts took place between July 30 and September 20, 2024. A subsequent evaluation phase was conducted from September 28 to November 22, 2024, to finalize the compilation of key scientometrics parameters and relevant health indicators.

### Eligibility criteria

Publications were included in the synthesis and analysis if they met the following criteria: (1) scientific articles published in peer-reviewed journals as part of a serial publication process; (2) full-text availability; and (3) a clearly stated objective focused on examining, discussing, investigating, synthesizing, or exploring the application or implementation of Six Sigma in healthcare.

Exclusion criteria encompassed: (1) conference proceedings, book chapters, books, errata, and retracted publications; (2) documents lacking essential bibliographic details, such as author information, journal name, or correspondence details; and (3) articles categorized as *in press*.

Publications originally released in languages other than English or Spanish were considered eligible if their abstracts were available in either of these languages and they fulfilled all inclusion criteria while avoiding any exclusion conditions. Given the historical nature of the study, no restrictions were imposed on the publication year of the included documents.

### Data standardization

Following data retrieval from multiple databases, the records were exported in .CSV format, incorporating all available metadata, including document titles, author names and institutional affiliations, keywords, publication year, citation count, publication type, and other relevant bibliographic details. A preliminary manual review was conducted by two researchers to remove duplicate entries and assess the titles and abstracts for adherence to the inclusion and exclusion criteria. This initial screening was performed using Microsoft Office Excel 2016.

A subsequent evaluation was carried out by two researchers to complete the extraction of key scientometrics variables, healthcare quality indicators, and global health metrics. Any discrepancies identified during this phase were resolved by a third reviewer. Additionally, data standardization procedures were applied to enhance uniformity across the dataset. For instance, all review articles, regardless of methodological design (e.g., narrative reviews, systematic reviews with or without meta-analysis), were classified under the category *reviews*. Similarly, letters to the editor, correspondences, notes, and commentaries were consolidated into the *letters* category. For the *country* variable, the corresponding author’s country of affiliation was used as the primary reference.

### Data synthesis and analysis

To assess scientometrics indicators, the quartile ranking and H-index of each publication (according to the journal in which it was published) were extracted and standardized according to the year of publication. This data was sourced from the historical archives of the *Scimago Journal & Country Rank* (available since 1999) and the *Journal Citation Reports* (available since 1997), selecting the most favorable metric corresponding to the journal where the study was published.

Countries were categorized based on income level, classified into low-income (LICs), lower-middle-income (LMICs), upper-middle-income (UMICs), and high-income groups (HICs). This classification adhered to the 2024 World Bank open-access criteria^19^, ensuring consistency with globally recognized economic stratifications.

Additionally, twenty-two indicators related to health, research, and expenditure were obtained using the World Bank^20^ API via the wbstats library^21^ in R software^22^. The definitions of each of the indicators can be found in the official web site. Three additional indicators (https://doi.org/10.5281/zenodo.14845596) were manually retrieved from the *Global Observatory on Health Research and Development* of the World Health Organization^23^. All indicator data, along with the total number of publications and the average H-index of the articles, were summarized by the four World Bank income groups and available years. This dataset served as the basis for subsequent analyses. The indicators and the letters denoting each one for analysis can be identified in Appendix II.

To investigate the associations between global health indicators and bibliometric variables, we applied multiple linear regression models. For each model, we estimated the regression coefficient β (represents the expected change in the dependent variable for each one-unit increase in the independent variable, holding all else constant; a positive β indicates a direct association, while a negative β reflects an inverse relationship), its standard error, the adjusted coefficient of determination R² (quantifies the proportion of variability in the dependent variable explained by the model, adjusted for the number of predictors included), and the p-value.

Two bibliometric metrics were used in the analysis: the number of Six Sigma-related publications and the average H-index of the journals in which these publications appeared. Each of these was examined both as a dependent and independent variable, depending on the research question being addressed. For example, in some models, the number of publications was the dependent variable and a health indicator (e.g., current health expenditure) was the predictor; in others, the health indicator (e.g., adult mortality rate) was the dependent variable and the number of publications was the predictor. This flexible modeling strategy allowed us to explore bidirectional associations between scientific output and global indicators. Details regarding variable assignments and model specifications for each analysis are provided in the supplementary material at https://doi.org/10.5281/zenodo.14845596. Separate models were constructed for each World Bank income group. Only models with statistically significant results and expected effect sizes are presented in the main text; complete results are provided in supplementary material.

To better understand the relationships between bibliometric indicators and health-related variables across countries, all regression coefficients were standardized using Z-scores (a Z-score represents the number of standard deviations a particular coefficient deviates from the mean of the distribution, allowing for direct comparison across variables that may differ in scale or unit; a Z-score >0 denotes a coefficient above the mean and stronger-than-average association, while a Z-score <0 indicates a coefficient below the mean and weaker-than-average or inverse association). These standardized coefficients were compiled into a matrix, with each row representing an income group and each column corresponding to a global health, research, or expenditure indicator. This transformation allowed us to assess the relative strength and direction of associations within and across income groups. The resulting matrix was analyzed using hierarchical clustering to detect patterns of similarity in the associations between indicators and Six Sigma research metrics. Euclidean distance was used to measure dissimilarity between clusters, and the complete linkage method was applied to determine groupings.

Finally, heatmaps were generated to visually represent the standardized regression coefficients. In these heatmaps, warmer colors (e.g., red) typically indicate stronger positive associations, while cooler colors (e.g., blue) reflect stronger negative associations. The use of Z-scores enables intuitive interpretation by highlighting which indicators have relatively stronger or weaker associations with Six Sigma research outputs across economic contexts.

To synthesize the associations between Six Sigma research metrics and global health indicators across income groups, we conducted a meta-analysis using the results of the linear regression models described previously. Specifically, for each health indicator, we extracted the β coefficients and their standard errors from the separate models built for each World Bank income group (HICs, UMICs, LMICs, and LICs). These regression coefficients were treated as individual effect sizes and combined in a random-effects meta-analytic model, which accounts for between-group variability and assumes that true effect sizes may vary across income contexts. The Restricted Maximum Likelihood (REML) method was used to estimate the between-group variance, allowing for more accurate inference. Each effect size was weighted by the inverse of its variance, meaning that coefficients with greater precision (i.e., smaller standard errors) had a greater influence on the overall pooled estimate. This weighting ensures that results from more robust models contribute proportionally more to the meta-analytic findings.

Only indicators showing statistically significant pooled associations are reported in the main manuscript; a complete set of meta-analysis results for all indicators, including forest plots and heterogeneity statistics, is available in the supplementary material.

All statistical analyses were conducted using R software (version 4.3.1)^24^. The scripts for these analyses, along with detailed annotations, are available in the supplementary material at https://doi.org/10.5281/zenodo.14845596

## RESULTS

### Six Sigma-related publications in healthcare: Trends and characteristics by World Bank income groups

**Figure 1:**
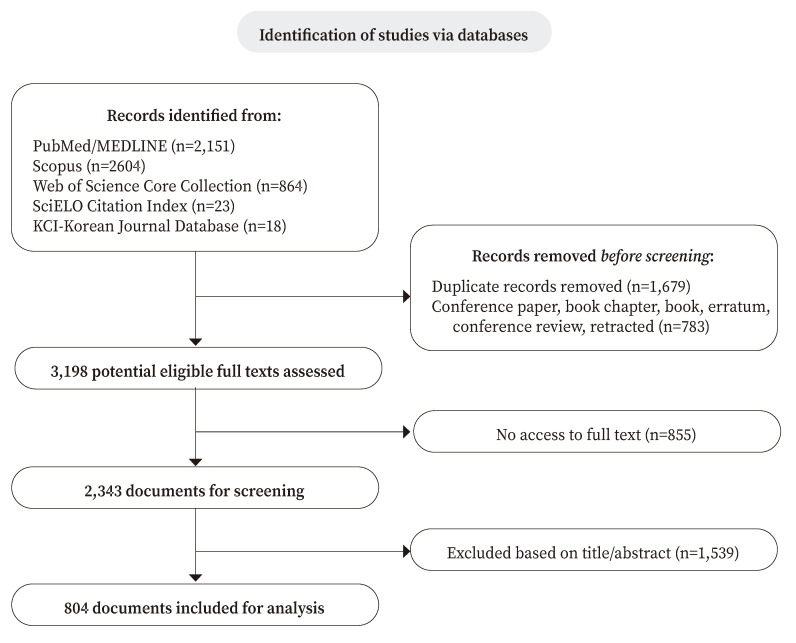
Documents selection flow diagram.

HIC: high-income countries; UMIC: upper-middle-income countries; LMIC: lower-middle-income countries; and LIC: low-income countries.

**Figure 2:**
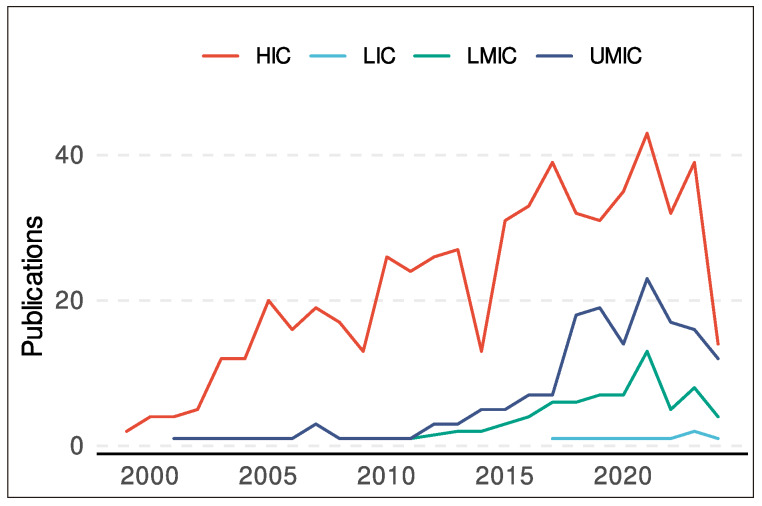
Annual trends in Six-Sigma-related publications in healthcare by World Bank income group (1999-2024).

HIC: high-income countries; UMIC: upper-middle-income countries; LMIC: lower-middle-income countries; and LIC: low-income countries.

A total of 804 Six Sigma-related publications in healthcare were included in this study (Figure 1), most of which (70.8%) originated from HICs. Notably, HICs was also the first group to publish on Six Sigma, with its inaugural Six Sigma-related publication appearing in 1999. In comparison, LMICs and LICs did not publish their first publication until 2008 and 2017, respectively (Figure 2).

Although LICs contributed only six articles overall, nearly all were published as open access, yielding an open-access ratio of 5. The only other group with an open-access ratio above 1 was UMICs, at 1.17. By contrast, in the remaining income groups, non-open access publications outnumbered open-access ones, with HICs showing the lowest open-access ratio (0.48). HICs also exhibited the highest average H-index, while LMICs and LICs recorded the lowest averages (Table 1).

For every income group, the most prevalent document type was *Article*, followed by *Review*. LICs had the highest proportion of articles, followed by LIMCs; the largest share of reviews was observed in HICs, followed by UMICs (Table 1).

Among the 783 articles with reported journal quartile classifications, HICs had the highest percentage of Q1 articles, whereas UMICs led in the proportion of Q2 articles. In contrast, LMICs had the smallest fraction of Q1 publications and the highest proportion of Q4 publications; meanwhile, HICs recorded the lowest proportion of Q4 articles (Table 1).

Although LIC’s total publication count was low, more than half of its articles appeared in Q1 or Q2 journals (Table 1).

**Table 1:** General characteristics of Six Sigma-related publications in healthcare (n= 804) by income groups

	Countries income level			
	High	Upper-middle	Lower-middle	Low
Publications, *n (%)*	569 (70.8)	159 (19.8)	70 (8.7)	6 (0.7)
H-index, *mean (SD)*	75.6 (59.01)	61 (58.8)	50.3 (59.2)	60.5 (58.9)
Open Access, *n (%)*	185 (32.5)	86 (54.1)	28 (40.0)	5 (83.3)
Document type, *n (%)*				
Article	460 (80.8)	130 (81.8)	61 (87.1)	6 (100)
Letter	29 (5.1)	10 (6.3)	2 (2.9)	0
Review	80 (14.1)	19 (11.9)	7 (10)	0
Journal quartile, *n (%)*				
Q1	198 (35.6)	33 (21)	2 (3.1)	2 (33.3)
Q2	201 (36.2)	56 (35.7)	27 (42.2)	2 (33.3)
Q3	110 (19.8)	44 (28)	20 (31.2)	1 (16.7)
Q4	47 (8.5)	24 (15.3)	15 (23.4)	1 (16.7)

Countries were categorized based on income level according to the 2024 World Bank open-access criteria^19^; the corresponding author’s country of affiliation was used.

**Figure 3:**
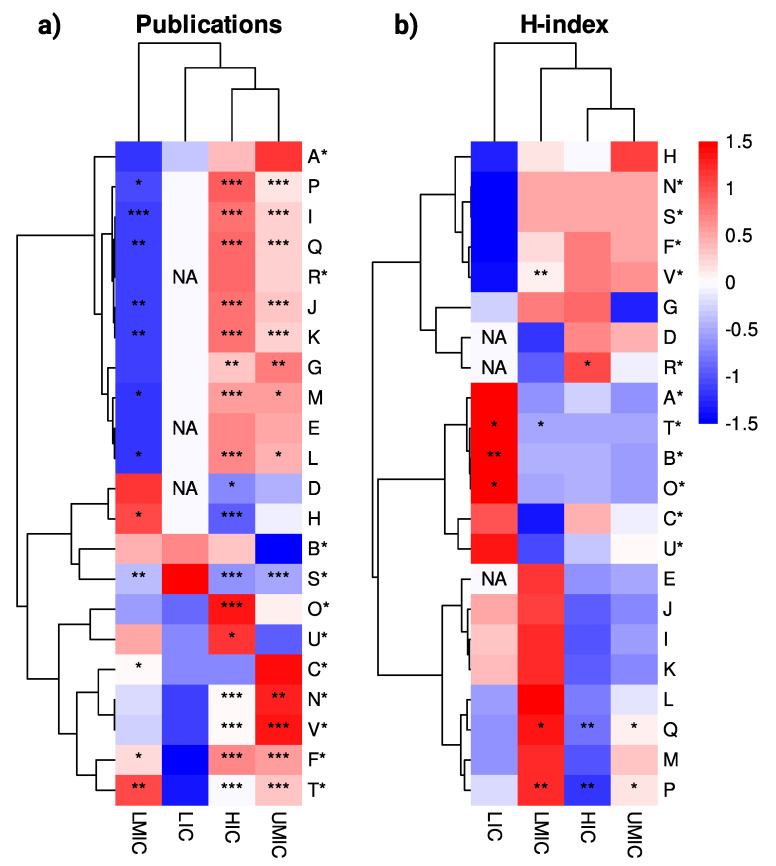
Heatmap of normalized linear regression coefficients (Z-scores) for the number of publications and average H-index, stratified by World Bank income groups. Indicators are represented by letters, with asterisks denoting those used as independent variables in the regression models.

HIC: high-income countries; UMIC: upper-middle-income countries; LMIC: lower-middle-income countries; and LIC: low-income countries. * p < 0.05; ** p < 0.01; *** p < 0.001.

Other correlations between scientometrics metrics and global health, research and development indicators are shown in Figure 3.

### High-income countries

In HICs, a strong inverse association was observed between the number of Six Sigma-related publications and adult mortality rates. Specifically, each additional publication was associated with 1.34 fewer deaths per 1,000 adult men (p < 0.001) and 0.69 fewer deaths per 1,000 adult women (p < 0.001). These associations were statistically significant and robust, with the models explaining approximately 79% and 76% of the variability in male and female mortality rates, respectively (p < 0.001 for both cases). These findings suggest that greater Six Sigma research output in HICs is linked to improved adult survival indicators.

Higher investment in research and development was positively associated with the number of Six Sigma-related publications. Specifically, a 1% increase in research and development expenditure was linked to 25.66 additional publications (p = 0.025), explaining approximately 21% of the variability in research output. Likewise, physician density had a strong positive association: for each additional physician per 1,000 people, the number of publications increased by 23.86 (p < 0.001), with this model accounting for nearly 76% of the variation. These findings suggest that greater investment in research and development and a larger medical workforce are important drivers of Six Sigma research productivity in well-resourced countries.

A larger specialist surgical workforce was significantly associated with higher journal quality, as measured by the H-index of the journals where Six Sigma-related studies were published. Specifically, each additional surgical specialist per 100,000 population was linked to a 0.65-point increase in the journal H-index (p = 0.012). This model explained approximately 68% of the variability, suggesting that countries with stronger surgical infrastructure tend to publish in higher-impact journals.

### Upper-Middle Income Countries

In UMICs, a significant inverse association was observed between the number of Six Sigma-related publications and adult mortality rates. Each additional publication was associated with 1.35 fewer deaths per 1,000 adult men (p = 0.013) and 1.10 fewer deaths per 1,000 adult women (p = 0.010). These associations explained approximately 31% and 33% of the variability in male and female mortality rates, respectively (p < 0.001). Although the associations were less strong than in HICs, the findings suggest that increased Six Sigma research activity in UMICs may also be linked to improved adult health outcomes.

Current health expenditure (CHE) was strongly associated with Six Sigma research output. Specifically, each 1% increase in CHE as a percentage of GDP was linked to 11.51 additional publications (p < 0.001). This relationship explained approximately 71% of the variability in research productivity, suggesting that greater investment in healthcare systems is a key driver of scientific output related to quality improvement initiatives in these countries.

### Lower-Middle Income Countries

In LMICs, an inverse association was also found between the number of Six Sigma-related publications and adult mortality rates. For females, each additional publication was associated with a reduction of 3.56 deaths per 1,000 adult women (p = 0.015), explaining about 43% of the variability. Among males, each additional publication was linked to 2.73 fewer deaths per 1,000 adult men (p = 0.040), accounting for approximately 33% of the variability. These findings suggest a potential link between increased Six Sigma research activity and improvements in adult survival, particularly among women, in LMICs.

In LMICs, CHE as a percentage of GDP was positively associated with the number of Six Sigma-related publications. Specifically, each 1% increase in CHE as a percentage of GDP corresponded to 9.47 additional publications (p = 0.039), explaining 33% of the variability in research output. Furthermore, ODA for medical research and basic health sectors per capita also showed a significant positive association: each additional dollar of ODA per capita was linked to 6.35 more publications (p = 0.032), accounting for 56% of the variability. These findings highlight the critical role of both domestic investment and international aid in fostering scientific productivity in quality improvement research within LMICs.

### Low-Income countries

No significant models were identified for LICs, likely due to the limited sample size in this category (only six publications).

### Impact of Six Sigma-related publications in healthcare in adult mortality rates

**Figure 4:**
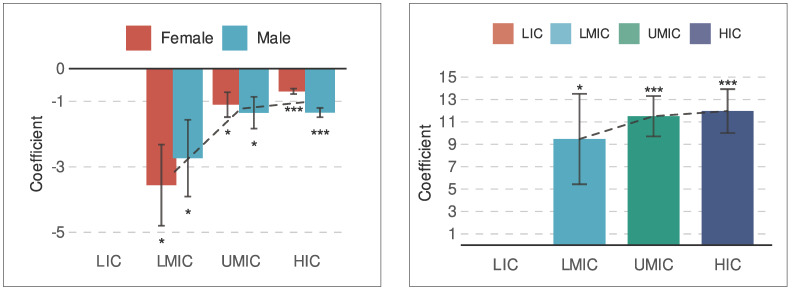
Coefficient (β) from linear models analyzing the volume of Six Sigma-related publications in healthcare across World Bank income groups in relation to adult male and adult female mortality rates (per 1,000 adults) as dependent variables (**A**) or current health expenditure (given as % of gross domestic product) as independent variable (**B**).

HIC: high-income countries; UMIC: upper-middle-income countries; LMIC: lower-middle-income countries; LIC: low-income countries. * p <0.05; ** p <0.01; *** p <0.001; dashed line: average coefficient across sexes (A) or comparison of results across categories (B).

Adult mortality rates (per 1,000 adults) for both males and females showed the strongest and most consistent associations with the number of Six Sigma-related publications across income groups. Therefore, we conducted a more focused analysis of these models. Results revealed a progressively stronger protective effect, from HICs to LMICs, indicated by increasingly negative β coefficients. This suggests that a higher volume of Six Sigma research is associated with lower adult mortality, particularly in better-resourced settings. In contrast, no meaningful associations were observed for LICs, likely due to the very limited number of relevant publications identified for this group (Figure 4A).

### Impact of current health expenditure (% of GDP) on six Sigma publication rates

CHE as a percentage of GDP showed a progressively stronger positive association with the number of Six Sigma-related publications when moving from LMICs to HICs. In each of these groups, a 1% increase in CHE was consistently associated with at least nine additional publications, indicating a significant and substantial relationship. Notably, CHE yielded the largest effect sizes among all independent variables analyzed, underscoring its critical role in driving research productivity in quality improvement. As with other indicators, no significant associations were detected for LICs, likely due to the very limited number of publications available (Figure 4B).

### Six Sigma publications in healthcare: Meta-analysis of associations with expenditure indicators

The meta-analysis combining regression results across income groups confirmed significant and protective associations between Six Sigma research output and key mortality indicators. Specifically, each additional publication was associated with an estimated reduction of 1.36 deaths per 1,000 adult men and 1.19 deaths per 1,000 adult women, suggesting a consistent inverse relationship between research activity and adult mortality. Similarly, the risk of catastrophic expenditure for surgical care, defined as the proportion of people at financial risk from surgical costs, also showed a protective association, with an estimated reduction of 0.43 percentage points per unit increase in publication volume (Table 2).

**Table 2:** Significant meta-analysis results of indicators used as dependent and independent variables in linear models related to number of publications

Mortality indicators	β	Weight
	(95%CI)	
* Research and development expenditure (% of GDP)	22.73	0.01
	(3.41 to 42.04)	
* Current health expenditure (% of GDP)	11.51	0.63
	(9.04 to 13.97)	
* Out-of-pocket expenditure per capita (current USD)	0.09	6854.68
	(0.03 to 0.14)	
% of people at risk of catastrophic expenditure for surgical care	-0.43	1078.33
	(-0.81 to -0.06)	
Adult female mortality rate/1,000 female adults	-1.19	153.41
	(-2.15 to -0.23)	
Adult male mortality rate/1,000 male adults	-1.36	54.88
	(-1.62 to -1.09)	

β: Beta coefficient; CI: confidence interval; *Used as independent variables in the model; GDP: gross domestic product; USD: United States dollar.

Regarding expenditure-related indicators, research and development investment had the strongest positive association with publication output. A 1% increase in research and development expenditure (as a percentage of GDP) was linked to an estimated increase of 22.73 publications. Additionally, CHE and out-of-pocket health spending per capita also showed significant positive associations with Six Sigma publication rates, with effect sizes of 11.51 and 0.09, respectively. These findings suggest that national investments in both research infrastructure and healthcare systems are strongly associated with scientific productivity in quality improvement research (Table 2).

## DISCUSSION

The findings of this study reveal distinct patterns in the distribution, impact, and evolution of Six Sigma research in healthcare across different income groups. HICs lead in research output, accounting for over 70% of Six Sigma-related publications. This dominance aligns with the higher levels of research and development expenditure, well-established healthcare systems, and robust academic infrastructures present in these countries^25^. The strong negative correlation between research output and adult mortality rates in HICs suggests that Six Sigma research may play a contributory role in improving healthcare outcomes by fostering process optimization and error reduction.

In contrast, UMIC and LMICs have demonstrated a gradual increase in Six Sigma research output over the past two decades, albeit at a slower pace than HICs. The significant association between CHE and research productivity in these groups may indicates that financial investment in healthcare is a key determinant of scientific output. However, the lower H-index values of the journals in which the publications appeared in LMICs suggest that while research volume is growing, its overall impact remains limited compared to HICs. This discrepancy underscores the need for greater international collaboration, funding mechanisms, and capacity-building initiatives to enhance research quality and dissemination in resource-constrained settings^26^.

LICs, on the other hand, exhibit minimal engagement in Six Sigma research, with only six publications identified over the study period. The lack of significant statistical associations in LICs is likely attributable to the small sample size and limited research infrastructure. Given that Six Sigma has the potential to improve efficiency in resource-limited healthcare systems, the near absence of research from LICs highlights a critical gap in the global knowledge landscape. Strengthening research capacity, increasing funding opportunities, and fostering partnerships with higher-income nations could facilitate the integration of Six Sigma methodologies into healthcare systems in LICs, ultimately contributing to improved healthcare quality and patient safety^27^^,^^28^.

From a policy perspective, the findings underscore the necessity of aligning Six Sigma research with global health priorities. Policymakers should consider incentivizing research on quality improvement methodologies, particularly in LMICs and LICs where healthcare inefficiencies and medical errors remain prevalent^29^. Additionally, integrating Six Sigma principles into national healthcare strategies could provide a structured framework for continuous quality improvement, leading to enhanced patient outcomes and more efficient healthcare delivery^30^.

Future research should explore the contextual factors influencing the adoption and success of Six Sigma in diverse healthcare environments. Expanding the scope of analysis to include implementation barriers, cost-effectiveness studies, and case-specific evaluations could provide deeper insights into the practicality of Six Sigma in different healthcare settings. Furthermore, fostering interdisciplinary collaborations between healthcare professionals, data scientists, and policymakers could drive innovation and accelerate the adoption of Six Sigma methodologies worldwide.

The methodological design of the study presents inherent limitations that should be considered when interpreting its findings. First, as a mixed-methods scientometrics study based on bibliographic databases, its reliance on the coverage and indexing quality of these sources may introduce selection bias, potentially omitting relevant research not published in journals indexed in consulted databases. Additionally, the classification of countries by income level, while useful for comparative analyses, does not capture the heterogeneity within each category, which may affect the interpretation of the identified associations. Furthermore, the use of regression models to assess correlations between Six Sigma research output and global health and development indicators does not allow for causal inference, limiting the ability to establish direct relationships.

In conclusion, this study provides a comprehensive overview of Six Sigma research in healthcare, highlighting its uneven global distribution and its correlation with key health and research indicators. The strong associations observed in HICs and UMICs suggest that Six Sigma methodologies may contribute to improving health outcomes and reducing inefficiencies in well-resourced settings. However, the low research engagement in LMICs and LICs signals an urgent need to address barriers such as limited funding, inadequate research infrastructure, and restricted access to scientific networks. Future studies should investigate the contextual factors influencing the adoption of Six Sigma in diverse healthcare systems, incorporating qualitative assessments of implementation challenges. Strengthening interdisciplinary collaboration between researchers, healthcare professionals, and policymakers will be essential to maximizing the impact of Six Sigma in global health and achieving sustainable improvements in patient care and safety.

## Data Availability

The dataset generated and analyzed is available and provided on request.

## APPENDICES

**Appendix I:** Search strategy in different databases or search engines

*Scopus*			
	SUBJAREA		TITLE-ABS
	heal	*AND*	“Six Sigma”
	*OR*		*OR*
	dent		“Total Quality Management”
	OR		*OR*
	*nurs*		“Continuous Quality Management”
	OR		*OR*
	medi		“Sigma Metric”
	*OR*		*OR*
	bioc		“Lean Six Sigma”
	*OR*		
	immu		
	*OR*		
	neur		
	*OR*		
	phar		
*PubMed*			
	[Title/Abstract]		
	“Six Sigma”		
	OR		
	“Lean Six Sigma”		
	*OR*		
	“Sigma Metric”		
	*OR*		
	“Total Quality Management”		
	*OR*		
	“Continuous Quality Management”		
*Web of Science Core Collection / SciELO Citation Index / KCI-Korean Journal Database*			
	TOPIC		
	“Six Sigma”		
	*OR*		
	“Lean Six Sigma”		
	*OR*		
	“Sigma Metric”		
	*OR*		
	“Total Quality Management”		
	*OR*		
	“Continuous Quality Management”		

**Appendix II:** Indicators used for regression models

	Indicator
*A*	Health researchers (in full-time equivalent), as a proportion of all researchers
*B*	Number of grants for biomedical research by funder, type of grant, duration and recipients (World RePORT)
*C*	Official development assistance (ODA) for medical research and basic health sectors per capita, by recipient country
*D*	Cause of death, by non-communicable diseases (% of total)
*E*	Cause of death, by communicable diseases and maternal, prenatal and nutrition conditions (% of total)
*F*	Current health expenditure (% of gross domestic product)
*G*	Death rate, crude (per 1,000 people)
*H*	Hospital beds (per 1,000 people)
*I*	Mortality rate, neonatal (per 1,000 live births)
*J*	Mortality rate, under-5 (per 1,000 live births)
*K*	Mortality rate, infant (per 1,000 live births)
*L*	Mortality rate, adult, female (per 1,000 female adults)
*M*	Mortality rate, adult, male (per 1,000 male adults)
*N*	Out-of-pocket expenditure per capita (current USD)
*O*	Physicians (per 1,000 people)
*P*	Risk of catastrophic expenditure for surgical care (% of people at risk)
*Q*	Risk of impoverishing expenditure for surgical care (% of people at risk)
*R*	Specialist surgical workforce (per 100,000 population)
*S*	Charges for the use of intellectual property, payments (balance of payments, current USD)
*T*	Charges for the use of intellectual property, receipts (balance of payments, current USD)
*U*	Research and development expenditure (% of gross domestic product)
*V*	Researchers in Research & Development (per million people)

USD: United States dollars.
